# Discrepancies in prevalence trends for HIV, hepatitis B virus, and hepatitis C virus in Haiphong, Vietnam from 2007 to 2012

**DOI:** 10.1371/journal.pone.0179616

**Published:** 2017-06-29

**Authors:** Azumi Ishizaki, Vuong Thi Tran, Cuong Hung Nguyen, Tomoaki Tanimoto, Huyen Thi Thanh Hoang, Hung Viet Pham, Chung Thi Thu Phan, Xiuqiong Bi, Thuc Van Pham, Hiroshi Ichimura

**Affiliations:** 1 Department of Viral infection and International Health, Graduate school of Medical Sciences, Kanazawa University, Kanazawa, Japan; 2 Hai Phong University of Medicine and Pharmacy, Hai Phong, Viet Nam; Centers for Disease Control and Prevention, UNITED STATES

## Abstract

We previously reported a significant reduction in the prevalence of human immunodeficiency virus type 1 (HIV) from 2007 to 2012 in people who inject drugs (PWID; 35.9% to 18.5%, *p* < 0.001) and female sex workers (FSW; 23.1% to 9.8%, *p* < 0.05), but not in blood donors (BD) or pregnant women, in Haiphong, Vietnam. Our aim in the present study was to assess trends in the prevalence of infection with hepatitis B and C viruses (HBV and HCV, respectively). We also investigated the coinfection rates of HBV and HCV with HIV in the same groups. Between 2007 and 2012, HBV prevalence was significantly decreased in BD (18.1% vs. 9.0%, *p* = 0.007) and slightly decreased in FSW (11.0% vs. 3.9%, *p* = 0.21), but not in PWID (10.7% vs. 11.1%, *p* = 0.84). HCV prevalence was significantly decreased in PWID (62.1% in 2007 vs. 42.7% in 2008, *p* < 0.0001), but it had rebounded to 58.4% in 2012 (2008 vs. 2012, *p* < 0.0001). HCV prevalence also increased in FSW: 28.6% in 2007 and 2009 vs. 35.3% in 2012; however, this difference was not significant (2007 vs. 2012, *p* = 0.41). Rates of coinfection with HBV and HCV among HIV-infected PWID and FSW did not change significantly during the study period. Our findings suggest that the current harm reduction programs designed to prevent HIV transmission in PWID and FSW may be insufficient to prevent the transmission of hepatitis viruses, particularly HCV, in Haiphong, Vietnam. New approaches, such as the introduction of catch-up HBV vaccination to vulnerable adult populations and the introduction of HCV treatment as prevention, should be considered to reduce morbidity and mortality due to HIV and hepatitis virus coinfection in Vietnam.

## Introduction

Hepatitis C virus (HCV), hepatitis B virus (HBV), and human immunodeficiency virus type 1 (HIV) share the same modes of transmission. As a consequence, coinfection of HCV and/or HBV with HIV is frequent, particularly in people who inject drugs (PWID) [1].

We previously reported that the prevalence of HIV decreased significantly from 2007 to 2012 in PWID (35.9% vs. 18.5%, *p* < 0.001) and in female sex workers (FSW; 23.1% vs. 9.8%, *p* < 0.05), while there was no significant change in the prevalence of HIV in pregnant women (0.5% in 2007 vs. 0.6% in 2009, *p* = 1.00) or blood donors (BD; 2.9% in 2007 vs. 1.0% in 2012, *p* = 0.29) in Haiphong, Northern Vietnam [2–4]. These reductions in HIV prevalence are due to the rapid expansion of combined antiretroviral treatment (cART) coverage in Vietnam, from 11% in 2005 to 28.4% in 2007 and 53.0% in 2012. These reductions were concurrent in this region with the expansion of harm reduction programs to prevent HIV transmission, such as clean needle and syringe programs, condom promotion programs, and methadone maintenance therapy for PWID and FSW [5]. As the mortality of HIV-infected individuals has decreased following the introduction of cART [6], the impacts of liver-related morbidity and mortality, particularly those due to coinfection with HBV and/or HCV, have increased [7, 8]. Thus, it is important to understand the epidemiology of HBV and HCV and to measure rates of coinfection with these viruses and HIV. Therefore, in the current study, we assessed the trends of HBV and HCV prevalence and rates of coinfection of HBV/HCV with HIV in PWID, FSW, pregnant women, and BD between 2007 and 2012 in Haiphong, Vietnam.

## Subjects and methods

In 2007, we recruited 760 male PWID (mean age ± standard deviation: 34.1 ± 7.5 years), 91 FSW (24.8 ± 6.0 years), 200 pregnant women (30.8 ± 9.3 years), and 210 BD (69 women, 140 men, and one unknown; 31.2 ± 10.3 years) [2, 9, 10]. In 2008, we recruited 302 male PWID (32.5 ± 8.5 years). In 2009, we recruited 63 FSW (30.1 ± 8 years), 166 pregnant women (26.1 ± 5.4 years), and 206 BD (24 women and 182 men, 29.1 ± 8 .3 years) [3]. In 2012, we recruited 389 PWID (32.2 ± 6.9 years), 51 FSW (29.8 ± 7.0 years), and 200 BD (39 women and 161 men, 29.7 ± 9.7 years) [4]. All participants resided in Haiphong, Vietnam and were newly recruited to the current study in each of the years listed above. None of the participants had received antiviral treatment for HIV, HBV, or HCV before recruitment. The ethical committees of Hanoi Medical University, Vietnam and Kanazawa University, Japan reviewed and approved the study protocols. We collected plasma samples from all participants after they had provided written informed consent. We tested the samples for anti-HIV antibodies (HIVAb) with DAINA SCREEN HIV 1/2 (Alere Medical Co., Ltd., Tokyo, Japan), HBV surface antigen (HBsAg) with DAINA SCREEN HBsAg II (Abbott Japan, Tokyo, Japan), and HCV core antigen (HCVAg) with ARCHITECT HCV Ag (Abbott Japan, Tokyo, Japan) and/or Lumispot Eiken HCV Antigen (Eiken Chemical, Tokyo, Japan). All of the tests were performed in accordance with the manufacturers’ instructions [2, 4, 9, 10]. Pairwise comparisons between the results obtained at different sampling periods were conducted with chi-squared and Fisher’s exact tests using the SPSS programs (IBMSPSS statistics 19, IBM Corporation, NY, USA). A *p* value of <0.05 was considered significant.

## Results

**Fig 1:**
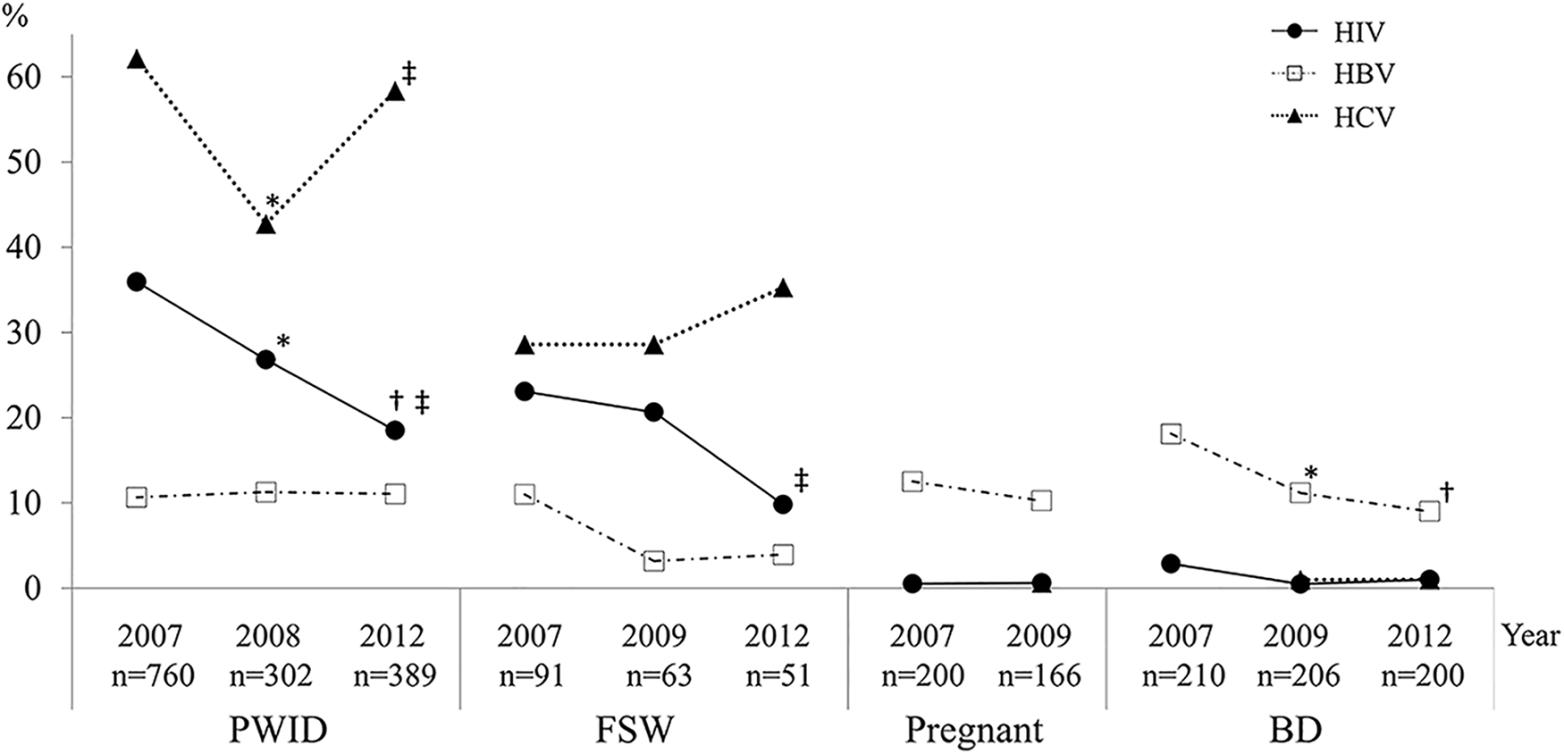
Changes in the prevalence of HIV, HBV, and HCV infection and coinfection in Haiphong, Vietnam from 2007 to 2012. Closed circle, HIV prevalence; open square, HBV prevalence; closed triangle, HCV prevalence. *, *p* < 0.05 for 2007 vs. 2008/9; †, *p* < 0.05 for 2007 vs. 2012; and ‡, *p* < 0.05 for 2008 vs. 2012. PWID, people who inject drugs; FSW, female sex workers; BD, blood donors.

From 2007 to 2012, the prevalence of HBV decreased significantly in BD, from 18.1% in 2007 to 11.2% in 2009 and 9.0% in 2012 (2007 vs. 2009, *p* = 0.046; 2009 vs. 2012, *p* = 0.47; 2007 vs. 2012, *p* = 0.007). HBV prevalence decreased slightly but not significantly in FSW, from 11.0% in 2007 to 3.2% in 2009 and 3.9% in 2012 (2007 vs. 2009, *p* = 0.12; 2009 vs. 2012, *p* = 1.00; 2007 vs. 2012, *p* = 0.21). The prevalence of HBV did not change significantly in PWID, from 10.7% in 2007 to 11.3% in 2008 and 11.1% in 2012 (2007 vs. 2008, *p* = 0.78; 2008 vs. 2012, *p* = 0.92; 2007 vs. 2012, *p* = 0.84), or in pregnant women, from 12.5% in 2007 to 10.2% in 2009 (*p* = 0.50) (Fig 1).

The prevalence of HCV decreased significantly in PWID, from 62.1% in 2007 to 42.7% in 2008 (*p* < 0.0001). However, it had rebounded in 2012 (58.4%) to a level similar to that of 2007 (2008 vs. 2012, *p* < 0.0001; 2007 vs. 2012, *p* = 0.22). In FSW, the prevalence of HCV did not change from 2007 (28.6%) to 2009 (28.6%), but it had slightly increased in 2012 (35.3%), although the difference was not significant (2007 vs. 2009, *p* = 1.00; 2009 vs. 2012, *p* = 0.44; 2007 vs. 2012, *p* = 0.41). HCV prevalence in BD did not change from 2009 to 2012 (1.0% in both years; 2009 vs. 2012, *p* = 1.00). Data regarding HCV prevalence in pregnant women were only available for 2009, when it was 0.6% (Fig 1).

There were no significant changes in rates of coinfection with HBV and/or HCV and HIV in PWID in 2007 (*n* = 273) and 2012 (*n* = 72). Coinfection rates in 2007 and 2012 were 2.9% and 5.6%, respectively, for HIV/HBV (*p* = 0.47); 81.3% and 83.3%, respectively, for HIV/HCV (*p* = 0.70); and 7.0% and 2.8%, respectively, for HIV/HBV/HCV (*p* = 0.27) (Table 1). Coinfection rates in FSW infected with HIV also did not change significantly between 2007 (*n* = 21) and 2012 (*n* = 5): 4.8% and 0%, respectively, for HIV/HBV (*p* = 1.00) and 71.4% and 80%, respectively, for HIV/HCV (*p* = 1.00). There were no triple infections in FSW throughout the study period (Table 1). Among BD infected with HIV (*n* = 6 in 2007 and *n* = 2 in 2012), only one individual was coinfected with HCV in 2012. There was no coinfection in HIV-infected pregnant women throughout the period.

**Table 1:** Trends in rates of coinfection with HBV and/or HCV and HIV in PWID and FSW from 2007 to 2012 in Haiphong, Vietnam.

	PWID			FSW		
Year	2007	2008	2012	2007	2009	2012
*n* (HIV infection)	273	81	72	21	13	5
HIV infection only	8.8%	16.1%	8.3%	23.8%	38.5%	20%
HIV and HBV coinfection	2.9%	4.9%	5.6%	4.8%	0%	0%
HIV and HCV coinfection	81.3%	75.3%	83.3%	71.4%	61.5%	80%
HIV, HBV, and HCV coinfection	7.0%	3.7%	2.8%	0%	0%	0%

There were no significant differences in coinfection rates in any groups throughout the observation period. PWID: people who inject drugs, FSW: female sex workers.

## Discussion

To the best of our knowledge, the present study was the first to investigate the trends of HIV, HBV, and HCV prevalence and coinfection rates in Vietnam. Previously, we reported that HIV prevalence decreased significantly in PWID and FSW, whereas it did not change significantly in BD between 2007 and 2012 in Haiphong, northern Vietnam [4]. In the current study, we used the same samples as in our previous study [4] and found that HBV prevalence did not change significantly in PWID during the study period, whereas it decreased sharply (although not significantly) in FSW from 2007 to 2009 (*p* = 0.12) and significantly in BD from 2007 to 2012 (*p* < 0.01). According to the Vietnam AIDS Response Progress Report, condom use in 2012–2013 was 92% in FSW and 41.2% in PWID [5]. In light of this report, the sharp reduction in HBV prevalence in FSW may be due to the consistent and widespread use of condoms in this group. The significant reduction of HBV prevalence in BD may be the consequence of efforts to replace paid and relative blood donations with unpaid volunteer blood donations and to increase the screening of donated blood for HBV [5].

The prevalence of HCV infection in PWID decreased significantly in 2008 compared to 2007 but then rebounded in 2012. In FSW, HCV prevalence did not change from 2007 to 2008 and then slightly increased in 2012. In Vietnam, 97.3% of PWID reported using sterile injecting equipment when they injected drugs last time, whereas only 20% of people who needed the treatment received the methadone maintenance therapy service in 2013 [5]. The clean needle and syringe program and methadone maintenance therapy could reduce the HCV prevalence among PWID [11] as observed from 2007 to 2008 in the current study. However, the HCV prevalence rebounded in 2012 to the level in 2007. This could be because the current harm reduction program has not yet reached to the majority of high-risk populations for HCV acquisition [12, 13] or may be insufficient to change their risky behaviors [14]. Our findings suggest that current approaches to control and prevent HIV infection, such as expansion of harm reduction programs for PWID and FSW, may be insufficient to reduce the prevalence of HCV in Vietnam.

We observed a wide range of coinfection rates with the hepatitis viruses among HIV-infected individuals in the different risk groups included in the current study. This finding is similar to that of previous reports of different risk groups and geographic areas in Vietnam. Previous studies reported the following rates of coinfection with HIV: HCV, 35.4–100%; HBV, 8.4–28%; and HBV/HCV, 0.5–5.6%. However, these previous studies used a different methodology to determine HCV infection; while we measured HCV antigen to confirm current HCV infection, previous studies used anti-HCV antibody, a marker for both past and current infection [15–18].

In high-HBV endemic areas such as Vietnam, the coinfection rates of HIV with HBV reflect the background prevalence of HBV in the general population [8,9,19]. HIV coinfection is associated with higher rates of HBV chronicity after acute infection, a lower rate of HBe antigen clearance, a higher frequency of cirrhosis, and a higher rate of liver-related mortality than infection with HBV alone [20]. Thus, it is important to prevent HBV acquisition in HIV-infected populations to reduce the burden of liver-related mortality due to HIV/HBV coinfection. Nationwide expansion of the expanded immunization program (EPI) on HBV for infants and the effort to enhance HBV vaccine coverage since 2002 have achieved a marked reduction in HBsAg prevalence from 3.64% in 2000 to 1.64% in 2008 among children under 5 years old in Vietnam [21]; however, the EPI has had no impact on adult population, such as those recruited in the current study. Implementation of catch-up HBV vaccination for vulnerable adult populations, such as people infected with HIV, PWID, and FSW, should be considered to reduce morbidity and mortality due to HIV/HBV coinfection [22, 23].

Several studies have shown that treating chronic HCV infection in PWID with new direct-acting antivirals (DAAs) is cost effective because it reduces the main reservoir of ongoing HCV transmission (HCV treatment as prevention) [24–27]. Durier et al. showed that introduction of HCV treatment for PWID could achieve a substantial reduction in HCV transmission and prevalence in Vietnam using a mathematical model [28]. Treatment of HCV in PWID and FSW coinfected with HIV should be considered to reduce HCV prevalence [29]. New comprehensive approaches, such as increased uptake of HCV testing, screening for HCV in people infected with HIV, introduction of HCV treatment as prevention, expansion of harm reduction programs with peer support, and augmented surveillance systems to address the risk factors associated with HCV acquisition, should be implemented to reduce the morbidity and mortality associated with HIV/HCV coinfection [30–32].

In conclusion, our findings suggest that the current harm reduction programs designed to prevent HIV transmission in PWID and FSW may be insufficient to prevent the transmission of hepatitis viruses, particularly HCV, in Haiphong, Vietnam. New approaches, such as the introduction of catch-up HBV vaccination to vulnerable adult populations and the introduction of HCV treatment as prevention, should be considered to reduce morbidity and mortality due to HIV and hepatitis virus coinfection in Vietnam.
